# Characterization of Medication Trends for Chronic Kidney Disease: Mineral and Bone Disorder Treatment Using Electronic Health Record-Based Common Data Model

**DOI:** 10.1155/2021/5504873

**Published:** 2021-11-22

**Authors:** Sungdam Han, Minkook Son, Byungjin Choi, ChulHyoung Park, Dong Ho Shin, Jong Hwan Jung, Min-Jeong Lee, Gyu-Tae Shin, Heungsoo Kim, Rae Woong Park, Inwhee Park

**Affiliations:** ^1^Department of Nephrology, Ajou University School of Medicine, 164, Worldcup-ro, Yeongtong-gu, Suwon, Gyeonggi-do 16499, Republic of Korea; ^2^Department of Biomedical Science and Engineering, Gwangju Institute of Science and Technology, 123, Cheomdangwagi-ro, Buk-gu, Gwangju 61005, Republic of Korea; ^3^Department of Biomedical Informatics, Ajou University School of Medicine, 164, Worldcup-ro, Yeongtong-gu, Suwon, Gyeonggi-do 16499, Republic of Korea; ^4^Department of Internal Medicine, College of Medicine, Hallym University, Kandong Sacred Heart Hospital, 150, Seongan-ro, Gangdong-gu, Seoul 05355, Republic of Korea; ^5^Division of Nephrology, Department of Internal Medicine, Wonkwang University School of Medicine and Hospital, 895, Muwang-ro, Iksan, Jeollabuk-do 54538, Republic of Korea; ^6^Department of Biomedical Sciences, Ajou University Graduate School of Medicine, 164, Worldcup-ro, Yeongtong-gu, Suwon, Gyeonggi-do 16499, Republic of Korea

## Abstract

Chronic kidney disease–mineral bone disorder (CKD-MBD) is the most common complication in CKD patients. Although there is a consensus on treatment guidelines for CKD-MBD, it remains uncertain whether these treatment recommendations reflect actual practice. Therefore, the aim of this study was to investigate the CKD-MBD medication trend in real-world practice. This was a retrospective and observational study using a 12-year period database transformed into a common data model from three tertiary university hospitals. Study populations were subjects initially diagnosed as CKD. The date of diagnosis was designated as the index date. New patients were categorized year to year from 2008 to 2019 with a fixed observation period of 365 days to check the prescription of CKD-MBD medications including calcium-containing phosphate binder, noncalcium-containing phosphate binder, aluminium hydroxide, vitamin D receptor activator (VDRA), and cinacalcet. The numbers of CKD patients in the three hospitals were 7555, 2424, and 5351, respectively. The proportion for patients with CKD-MBD medication prescription decreased yearly regardless of hospital and CKD stage (*p* for trend < 0.05). The use of aluminium hydroxide disappeared steadily while the use of VDRA increased annually in all settings. Despite these changes in prescription patterns, the mean value for CKD-MBD-related serologic markers was almost within target range. The proportion of the population within the target value was not significantly changed. Irrespective of hospital and CKD stage, similar trends of prescription for CKD-MBD medications were observed in real-world practice. Further research with a distributed network study may be helpful to understand medication trends in CKD-MBD treatment.

## 1. Introduction

Chronic kidney disease–mineral bone disorder (CKD-MBD) is the most common complication in CKD patients. Vascular calcifications, biochemical abnormalities, and bone abnormalities constitute the CKD-MBD syndrome. Uncontrolled CKD-MBD causes serious changes, potentially disabling the body with complications including parathyroid hyperplasia, bone pain, fractures, vascular calcification, and even cardiovascular problems [1]. Furthermore, CKD-MBD is ultimately associated with increased mortality [2]. Management of CKD-MBD has changed significantly over the past 20 years. In the 1990s, calcium-containing phosphate binders and calcitriol were increasingly used in the management of CKD-MBD. Due to concerns about hypercalcemia and oversuppression parathyroid hormone (PTH), a synthetic vitamin D analogue (paricalcitol), a noncalcium-containing phosphate binder (sevelamer), and a calcimimetic (cinacalcet) have entered the market since 1998, 1998, and 2004, respectively.

Kidney Disease Improving Global Outcomes (KDIGO) is a nonprofit foundation established in 2003 for patients with kidney diseases. It provides treatment strategies for patients with chronic kidney disease. KDIGO published the 2017 guidelines for CKD-MBD, an update of the guidelines first published in 2009 [3, 4]. A randomized controlled study in 2012 showed that when noncalcium-containing phosphate binders were administered to patients before dialysis, survival benefits were found compared to calcium-containing phosphate binders [5]. In addition, in a study conducted on hemodialysis patients, the risk of death from arrhythmia and cardiovascular disease was significantly lower in patients taking noncalcium-containing phosphate binders than in patients using calcium-containing binders [6]. Based on these results, the 2017 KDIGO guidelines recommended limiting the use of calcium-containing phosphate binders to control hyperphosphatemia in all adult patients at CKD stages 3a to 5 [3, 7]. The guidelines also recommended that CKD-MBD treatment strategies should be determined based on calcium, phosphate, and PTH concentrations in patients with CKD stages 3a to 5. They suggested that treatment should not be decided based on the results of a single test but should be determined based on results of regular measurements. As a result of analyzing a large cohort of dialysis patients, alcium, phosphate and PTH concentrations correlate and influence each other, so a treatment strategy tailored to each patient is needed [8]. The KDIGO guidelines are the most commonly used guidelines in the world, including in Korea.

Although there is a consensus on treatment guidelines, whether treatment recommendations reflect actual practice and whether there is any difference between actual practice and recommendations remain unclear. To identify these real-world prescription patterns, we used a common data model (CDM) that could allow for a systematic analysis of disparate observational databases [9]. Assessment of current prescription options for CKD-MBD using CDM in multiple hospitals will provide real evidence of common practice and treatment patterns. Therefore, the aim of this study was to show the applicability and feasibility of CDM in big data analysis for CKD-MBD. The primary goal of our study is to find out how well the current treatment pattern is consistent with the 2017 KDIGO guidelines. We also aimed to provide detailed information on the prescribing patterns of CKD-MBD and the differences between CKD stages and hospitals with changes in treatment trends from 2008 to 2019.

## 2. Materials and Methods

### 2.1. Data Source and Study Population

This was a retrospective and observational study using a 12-year period database from three tertiary university hospitals. Data were transformed with Observational Medical Outcomes Partnership (OMOP) CDM, version 5.3, by the Observation Health Data Sciences and Informatics (OHDSI) network (https://www.ohdsi.org/) [10]. The concept of CDM was to transform data into common representation (terminologies, vocabularies, and coding schemes) and common format (data model) and to perform systematic analyses using a library of standard analytic tools [9, 11]. It supports the access of multidatabase studies and translation of evidence across the individual hospitals and settings. The OMOP CDM applies standard vocabularies to normalize the meaning of data, such as Standard Nomenclature of Medicine (SNOMED) to represent clinical data, RxNorm to represent drugs, and Logical Observation Identifiers Names and Code (LOINC) to represent clinical measurements [12]. The CDM and open-source tools are opened on OHDSI GitHub (https://github.com/OHDSI/), and discussions are opened on a dedicated open forum (https://forums.ohdsi.org/). More information about OHDSI and the analytical pipelines is described in *The Book of OHDSI* [13].

We performed two main analyses with different study populations. First, we analyzed the CKD-MBD prescription pattern of CKD patients from three distinct hospitals. The study populations included subjects initially diagnosed as CKD with any SNOMED clinical terms that matched to N18.0, N18.1, N18.2, N18.3, N18.4, N18.5, N18.9, N19, Z49, Z94.0, or Z99.2 of the ICD-10 code [14–16]. The date of diagnosis was designated as the index date. Subjects were limited to those with age greater than or equal to 20 years. To investigate the trend for prescription of CKD-MBD drugs, new patients were categorized year to year from 2008 to 2019 with a fixed observation period of 365 days. Second, to further investigate the prescription pattern according to the CKD stage, subjects with the estimated glomerular filtration rate (eGFR) in one hospital database were categorized. The eGFR was calculated using the modification of diet in the renal disease equation [17]. To avoid counting CKD patients repeatedly and to evaluate medication pattern for the first diagnosis of CKD, we only include patients having the first diagnosis of CKD with at least one measurement of eGFR between 30 days before and 30 days after the index date. In addition, end-stage kidney disease (ESKD) patients with dialysis were subclassified and further analyzed. Details about cohort definitions and concept set definitions including diseases, procedures (SNOMED), measurements (LOINC), and medications (RxNorm) are described in Supplementary Material Appendixes 1 and 2.

### 2.2. Characterization for Study Population

We used the ATLAS program, which is a free, public, and web-based software developed by the OHDSI community that helps the design and analysis with standard, patient-level, and observational CDM format data [18]. In the ATLAS program, database-level characterization can be automatically performed, including a description of age, medical history, and medication use. In addition to baseline characteristics, the Charlson comorbidity index (CCI) was calculated based on the underlying disease, including myocardial infarction, congestive heart failure, peripheral vascular disease, cerebrovascular disease, dementia, chronic pulmonary disease, connective tissue disease, peptic ulcer, mild liver disease, diabetes with and without complications, paraplegia or hemiplegia, renal disease, any or metastatic cancer, moderate or severe liver disease, and acquired immune deficiency syndrome before the start of the follow-up period [19].

### 2.3. CKD-MBD-Related Medications

CKD-MBD-related medications were categorized as calcium-containing phosphate binder (calcium carbonate and calcium acetate), noncalcium-containing phosphate binder (sevelamer and lanthanum), aluminium hydroxide, vitamin D receptor activator (VDRA), and cinacalcet. The percentage of prescriptions for CKD-MBD-related medications was subsequently estimated yearly.

### 2.4. Open-Source Treatment Pathway

In this study, the treatment pathway in OHDSI [20], an open-source software with ATLAS, was used. For each patient in the cohort, this program identifies the sequence of medications in the RxNorm ingredient level, arranging them by first exposure to medications. If one CKD patient has at least one CKD-MBD-related medication, this patient is included in the group of persons with the treatment pathway. Conversely, the other CKD patient has no CKD-MBD-related medications; this patient is included in the group of persons without the treatment pathway. After each patient's sequence of medications was built, the number of patients with the same treatment sequence was counted and expressed by sunburst plots. These sequences were limited to 10 medications.

### 2.5. Calcium, Phosphate, and Parathyroid Hormone

Serum concentrations of calcium, phosphate, and albumin were determined with automated and standardized methods. Intact PTH (iPTH) concentration was measured with second-generation PTH assays at the hospital. Final laboratory values of calcium, phosphate, albumin, and iPTH within the follow-up period for CKD stage 5 patients were extracted. Corrected calcium was calculated with albumin as Equation (1) when the albumin level was less than 4.0 g/dL. Target ranges for this study were as follows: calcium, 8.6 to 10.2 mg/dL; serum phosphate, 2.5 to 4.5 mg/dL; and iPTH, 130 to 600 pg/mL. These were recommended by the 2017 CKD-MBD KDIGO guidelines and a multicentre study in Korea [3, 21, 22].

(1) $$\begin{array}{c} \text{Corrected calcium}=\text{Total calcium}+0.8\times \left(4.0-\text{Albumin}\right). \end{array}$$

### 2.6. Analytical and Statistical Methods

This was a descriptive study for CKD-MBD medications according to the hospital and CKD stage. Continuous variables are presented as mean and standard deviation. Categorical variables are expressed as number and percentage. To refine and analyze data, Structured Query Language and R programming language version 4.1.0 (R foundation for Statistical Computing, Vienna, Austria) were implemented. To compare the characteristics of CKD patients for the three different hospitals, the one-way analysis of variance and the chi-square test were performed. To evaluate the prescription trend in the different hospitals and CKD stages, the Cochran-Armitage trend test was performed. Statistical analyses were performed with R version 4.1.0. The *p* value < 0.05 was considered statistically significant.

### 2.7. Ethical Consideration

Since all databases transformed into CDM were deidentified, this study was approved by the Institutional Review Board of Ajou University Hospital (approval no. AJIRB-MED-MDB-21-241) with a waiver for informed consent.

## 3. Results

### 3.1. Baseline Characteristics of Subjects

Table 1 shows the baseline characteristics of subjects from three different hospitals. The numbers of CKD patients in the three hospitals were 7555, 2424, and 5351, respectively. The proportion of those over 80 years of age was higher in the B and C hospitals (19.83% and 20.73%, respectively) than in the A hospital (13.42%) with a significant *p* value (<0.001). Males accounted for 58% to 60%. Subjects from the A hospital had more diabetes and CKD stage 5. Subjects from the C hospital had more dyslipidemia and cerebrovascular disease with a higher Charlson comorbidity index (all *p* values < 0.001). Regarding medication use, subjects from the B hospital had more medication prescriptions except for opioids and corticosteroids. Overall, there were significant differences in baseline characteristics from three different hospitals except for sex.

### 3.2. Yearly CKD-MBD Medication Prescription Pattern in CKD Patients from Three Different Hospitals

**Figure 1:**
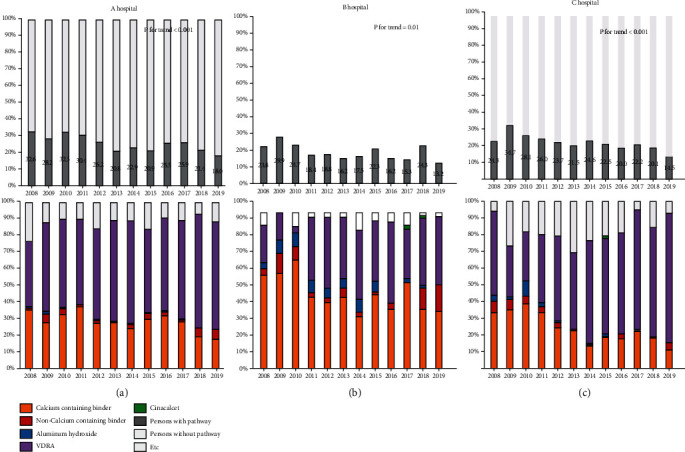
Yearly CKD-MBD medication prescription patterns for CKD patients from three different hospitals. Abbreviations: CKD-MBD: chronic kidney disease-mineral bone disorder; VDRA: vitamin D receptor activator.

Yearly prescription patterns in CKD patients from 3 different hospitals are shown in Figure 1. Although proportions of CKD-MBD medication prescriptions were different among the three hospitals, these proportions decreased yearly (all *p* for trend <0.05). Proportions of CKD-MBD medication prescriptions in 2019 were roughly half of those in 2008 in all hospitals. Regarding classes of CKD-MBD medications, the percentage of calcium-containing phosphate binder decreased dramatically from 2008 to 2019 (all *p* for tend <0.05), while the percentage for noncalcium-containing phosphate binder increased gradually from 2018. The use of aluminum hydroxide disappeared steadily while the use of VDRA increased annually (all *p* for trend < 0.05). These patterns of prescription were found for all hospitals.

### 3.3. Yearly CKD-MBD Medication Prescription Pattern for CKD Patients according to CKD Stage

**Figure 2:**
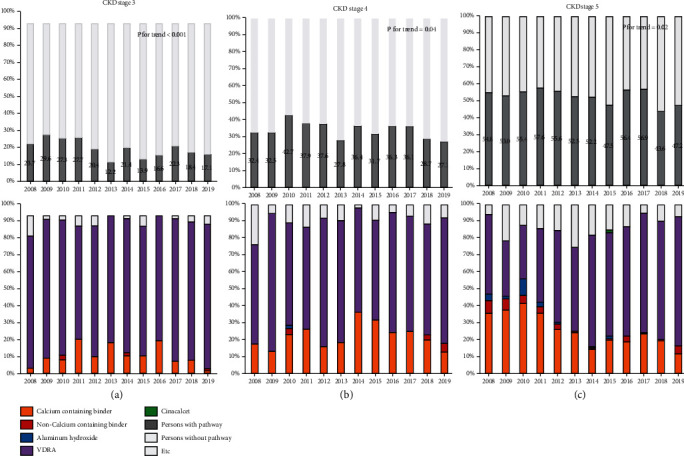
Yearly CKD-MBD medication prescription patterns for CKD patients according to CKD stage. Abbreviations: CKD-MBD: chronic kidney disease-mineral bone disorder; VDRA: vitamin D receptor activator.

Yearly prescription patterns for CKD patients according to the CKD stage with the diagnosis code and eGFR in the A hospital are shown in Figure 2. The numbers of CKD patients according to the CKD stage were 4154 for CKD stage 3, 1622 for CKD stage 4, and 1779 for CKD stage 5. Proportions of CKD-MBD medication prescriptions decreased yearly regardless of CKD stage (all *p* for trend <0.05). In particular, reduced fractions of prescriptions from 2008 to 2019 were as follows: 28% for CKD stage 3, 17% for CKD stage 4, and 14% for CKD stage 5. For CKD stage 3, the percentage of calcium-containing phosphate binder was 1.4% in 2019. For CKD stages 4 and 5, percentages of calcium-containing phosphate binder tended to decrease over time but were statically nonsignificant (*p* for trend = 0.50 and 0.08, respectively). The use of aluminum hydroxide disappeared while the use of VDRA increased over time for those with CKD stage 5 (all *p* for trend < 0.05). The proportion for CKD-MBD medication in ESKD patients with dialysis also decreased from 2008 to 2019. The prescription pattern for ESKD patients with dialysis is described in Supplementary Figure S1.

### 3.4. Treatment Pathway for CKD-MBD Medication according to CKD Stage

**Figure 3:**
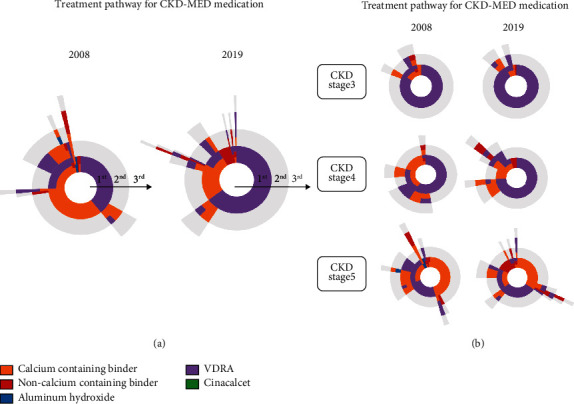
Treatment pathway for CKD-MBD medication according to CKD stage. Abbreviations: CKD-MBD: chronic kidney disease-mineral bone disorder; VDRA: vitamin D receptor activator.

The treatment pathway for CKD-MBD medication according to the CKD stage is shown in Figure 3. Regarding the first prescription of CKD-MBD medication, the percentage of calcium-containing phosphate binder decreased while the percentage of noncalcium-containing phosphate binder increased from 2008 to 2019 (all *p* for trend < 0.05). In particular, the prescription of the noncalcium-containing phosphate binder appeared as a secondary or tertiary prescription in 2008 but appeared increasingly as the first prescription in 2019. The use of aluminum hydroxide disappeared while the use of VDRA increased from 2008 to 2019 (all *p* for trend < 0.05). The treatment pathway for CKD-MBD medication in ESKD patients with dialysis is shown in Supplementary Figure S1.

### 3.5. CKD-MBD-Related Serologic Markers of CKD Stage 5 Patients in Hospital A

**Figure 4:**
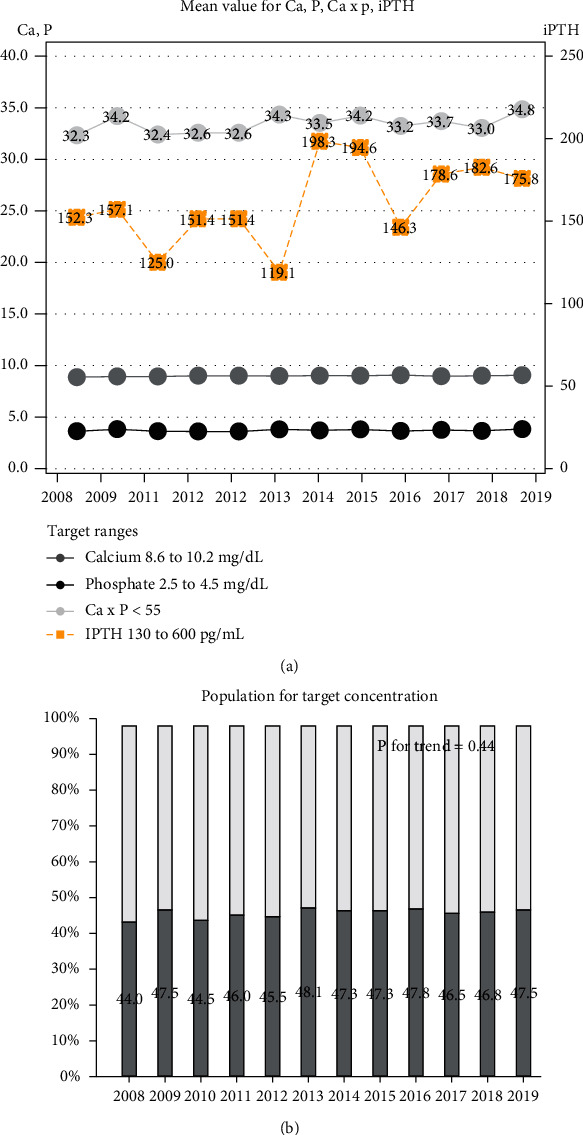
CKD-MBD-related serologic markers in patients with CKD at stage 5. Target levels: calcium, 8.4 to 9.6 mg/dL; phosphate, 2.4 to 5.0 mg/dL; Ca×P product, <55 mg^2^/dL^2^; and iPTH, 100 to 300 pg/mL. Abbreviations: CKD-MBD: chronic kidney disease-mineral bone disorder; iPTH: intact parathyroid hormone.

To evaluate the effect of mutational trends of CKD-MBD medication, values of calcium, phosphate, calcium×phosphate, and iPTH in CKD stage 5 patients were investigated in hospital A. CKD-MBD-related serologic markers are shown in Figure 4. Mean values of calcium×phosphate product were 32.3 to 34.8 mg/dL (standard deviation (SD) 5.7 to 7.0). Mean values of iPTH were 119.1 to 198.3 pg/mL (SD 72.5 to 295.2). The proportion of the population within the target value ranged from 44.0% to 48.1%, showing no significant change from 2008 to 2019 (*p* for trend > 0.05).

## 4. Discussion

This study evaluated the pattern of CKD-MBD medication using the CDM database from three different hospitals. Approximately, the proportion of patients with CKD-MBD medication prescription decreased yearly regardless of hospital and CKD stage. The use of aluminum hydroxide disappeared steadily while the use of VDRA increased annually in all settings. Despite these changes in prescription patterns, mean values of CKD-MBD-related serologic markers were within their target ranges and the proportion of the population within these target values was not significantly changed.

Patients from these three hospitals showed different baseline characteristics. There were differences in the proportion of patient age, past medical history, CKD stage, and medication history. According to the Korean Renal Data System registry results for 2019 [23], diabetes accounts for about 50% of ESKD patients, and these fractions are very different from the above baseline characteristics. Such differences among these three hospitals have an influence on the community where these hospitals are located. In addition, it seemed that there was a difference in the behavior of entering diagnosis codes for each hospital.

According to the results of our study on drug prescription proportion, there were differences between hospitals. However, the overall prescription proportion decreased. Several reasons can be considered for such decrease. First, this decrease could be attributable to an increase in overall CKD prevalence [24]. According to data released by the Korean National Health Insurance Service in 2015, the prevalence of CKD patients increased by 13.6% between 2009 and 2013. Causes include an increase in the elderly population and an increase in the number of chronic diseases such as hypertension and diabetes. Another cause might be early detection of CKD patients through screening. About 15% of newly increased patients with CKD were treated at tertiary university hospitals [25]. Among them, the patients who did not need CKD-MBD treatment were included, which might have led to our study results. Second, the prescription was reduced by establishing nutrition education such as a phosphate restriction diet. Several studies have reported that a phosphate restriction diet is helpful for hyperphosphatemia [26]. Despite some limitations, the KDIGO 2017 phosphate restriction diet is useful for treating hyperphosphatemia. Third, in 2009 and 2017, the KDIGO CKD-MBD guideline was published [3, 4]. It was thought that the use of unnecessary prescription for CKD-MBD was reduced through evidence-based medicine.

In South Korea, CKD is regarded as one of the most significant health problems with the highest cost per patient [27]. All South Korean citizens were covered by the national health insurance (NHI), which reviews most of medical care including medication. For CKD-BMD prescription, although the NIH covers the use of noncalcium-containing phosphate binder to only ESKD patients as shown in the results of this study, the proportion of those using noncalcium-containing phosphate binder is gradually increasing at all CKD stages. It appeared to be increasingly used as the first medication. This seems to be the result of physicians choosing medications according to the 2017 KDIGO CKD-MBD guidelines even if insurance issues are taken into account. In 2018, the NIH relaxed the insurance coverage for noncalcium-containing phosphate binder. However, it still limited the treatment target to ESKD patients.

As shown in this study, the use of aluminum hydroxide for limiting phosphate absorption was deprecated. Long-term use of aluminum hydroxide is known to be associated with side effects such as osteomalacia and encephalopathy caused by aluminum. Its use seems to have gradually decreased accordingly [28]. PTH elevation may represent a compensatory response to hyperphosphatemia and increasing bone resistance to PTH. Moreover, in two past randomized trials of VDRA in CKD patients, the use of VDRA showed a decrease in PTH but no change in cardiovascular end points with an increased risk of hypercalcemia [29, 30]. For these reasons, the 2017 KDIGO CKD-MBD guidelines recommend that VDRA should not be routinely used for those with abnormal PTH levels. However, in our study, VDRA use showed a gradual increase. In a previous study, since vitamin D deficiency is common in CKD patients [31, 32], it is thought that the prescription of nutritional vitamin D (cholecalciferol and ergocalciferol) has increased. In this study, since VDRA was investigated as both active vitamin D and nutritional vitamin D, further studies are needed in the future. In spite of changing treatment trends according to guidelines, in our study, the proportion of patients included in the treatment target did not differ significantly from 2008 to 2019. Of course, since the use of noncalcium-containing phosphate binder is associated with survival benefits and reduced risk of death from arrhythmia and cardiovascular disease [5, 6], laboratory tests alone cannot determine the usefulness guideline. However, several studies have recently shown no significant difference in composite cardiovascular events between the use of calcium-containing phosphate binder and noncalcium-containing phosphate binder [33, 34]. In terms of medical costs, a noncalcium-containing phosphate binder costs nearly 20–30 times more than a calcium-containing phosphate binder, which is a significant burden on both the NIH and individual patients. Given the growing trend in the use of noncalcium-containing phosphate binder, its benefits need to be further elucidated.

In this study, CDM-based analysis suitable for big data analysis was used. With CDM, we could perform analyses in the distributed region and include a large number of CKD patients. If the traditional method of reviewing medical records was used, it would be very laborious and subject to human errors. This study confirmed the applicability and feasibility of CDM-based analysis and verified the possibility of using it for a future distributed network study in the field of nephrology. However, this study has some limitations. First, because a deidentified database was used in this study, we could not review the medical records of individual patients or consider their specific situations. Second, although the exact diagnosis for CKD was necessary, we used diagnostic codes based on the definition of CKD. This definition issue is a common problem in CDM-based analysis. Therefore, we used the CKD definition, which was used in previous studies [14–16]. Third, to investigate the pattern of prescription according to CKD stage, subclassification of CKD stages using eGFR values was performed, but subgrouping was performed only in the A hospital. In addition, individual for CKD stages can be varied during the follow-up period. However, according to a 10-year observational study of Asian CKD patients in the past, the rate of eGFR decline in the participants was 0.36 mL/min/1.73 m^2^/year on average [35]. The number of patients whose CKD stage has changed over the course of one year is thought to be small. Fourth, the association between reduced use of calcium-containing phosphate binder and cardiovascular outcomes was not investigated. Finally, we enrolled patients from three different hospitals, but we cannot extract the laboratory data in all hospitals. This is the common problem using CDM due to the limitation of the current version of OHDSI CDM. Considering these limitations, further external validation is needed to confirm our results.

## 5. Conclusions

In summary, this is the first study to investigate prescription patterns of CKD-MBD in Korea using CDM. The proportion of patients with CKD-MBD medication prescription decreased yearly regardless of hospital and CKD stage. However, mean values for CKD-MBD-related serologic markers were not significantly changed for the study period. Based on results of this study, further research with a distributed network study may be helpful to understand medication trends in CKD-MBD treatment.

## Figures and Tables

**Table 1:** Baseline characteristics of subjects from three different hospitals.

Baseline characteristics	A hospital (*n* = 7555)	B hospital (*n* = 2424)	C hospital (*n* = 5351)	*p* value
Age group (%)				
20–24	<1	<1	<1	NA^†^
25–29	<1	<1	<1	
30–34	1.51	<1	1.18	
35–39	2.33	2.06	2.11	
40–44	3.87	2.35	2.63	
45–49	6.17	4.00	4.18	
50–54	8.86	6.60	5.49	
55–59	9.48	9.60	8.83	
60–64	10.84	10.14	10.20	
65–69	12.10	13.03	11.75	
70–74	15.02	15.25	14.51	
75–79	14.03	15.05	16.19	
80–84	9.73	11.01	13.05	
Over 85	3.69	8.82	7.68	
Sex (%)				0.14
Male	60.54	59.93	58.79	
Female	39.46	40.07	41.21	
Medical history (%)				
Hypertension	52.68	41.47	60.3	<0.001
Diabetes	31.06	17.89	26.85	<0.001
Dyslipidemia	6.32	5.56	20.5	<0.001
Cerebrovascular disease	9.17	8.45	14.98	<0.001
Heart failure	5.12	7.46	5.08	<0.001
CKD stage 5^‡^	14.29	6.64	8.66	<0.001
CKD stage 4^‡^	8.52	7.75	12.94	<0.001
CKD stage 3^‡^	3.29	24.32	30.77	<0.001
Charlson comorbidity index, mean (SD)	4.06 (2.30)	4.15 (1.94)	4.40 (2.27)	<0.001
Medication use (%)				
Agents acting on the renin-angiotensin system	50.06	61.17	58.21	<0.001
Beta-blocking agents	36.70	48.43	41.98	<0.001
Calcium channel blockers	50.42	61.29	56.53	<0.001
Diuretics	47.20	63.36	59.2	<0.001
Antidiabetic drugs	39.96	53.71	47.58	<0.001
Lipid-modifying agents	59.26	71.39	61.61	<0.001
Antithrombotic agents	52.24	67.31	61.62	<0.001
Opioids	57.67	62.94	63.96	<0.001
Anti-inflammatory and antirheumatic products	65.25	76.59	76.06	<0.001
Corticosteroids	37.81	39.86	43.47	<0.001

^†^To protect individual data, results under the 1% cannot be calculated in the ATLAS program. Chi-square test for the age group cannot be performed. ^‡^In a table, CKD stage was categorized by only Standard Nomenclature of Medicine (SNOMED) diagnosis code (CKD stage 5: 443611; CKD stage 4: 443612; CKD stage 3: 443597). Abbreviations: CKD: chronic kidney disease; NA: not applicable; SD: standard deviation.

## Data Availability

The data used during the present study are available from the corresponding author upon reasonable request.

## Supplementary Materials

Supplementary Materials Appendix 1: cohort definition. Appendix 2: concept set definitions. Figure S1: yearly CKD-MBD medication prescription pattern and treatment pathway for ESKD patients.
