# Supplementary material for: Characterization of Medication Trends for Chronic Kidney Disease: Mineral and Bone Disorder Treatment Using Electronic Health Record-Based Common Data Model
Source: Biomed Res Int. 2021 Nov 22;2021:5504873. doi: 10.1155/2021/5504873 (PMC8629641; doi:10.1155/2021/5504873)
Supplement: Supplementary Materials — Appendix 1: cohort definition. Appendix 2: concept set definitions. Figure S1: yearly CKD-MBD medication prescription pattern and treatment pathway for ESKD patients. [file 5504873.f1.docx]

**Supplementary Material Appendix 1: Cohort definition**

**1. Patients with chronic kidney disease**

People who have the following:

- Condition occurrence of 'chronic kidney disease' who are ≥ 20 years old.
  - For the first time in the person's history.

with continuous observation of at least 180 days prior and 0 day after event index date, and limit initial events to the **earliest event per person.**

The cohort end date will be offset from the index event's start date plus 365 days.

**2. Patients with chronic kidney disease stage 3**

People who have the following:

- Condition occurrence of 'chronic kidney disease' who are ≥ 20 years old.
  - For the first time in the person's history.
- having at least one measurement of 'MDRD GFR'
  - starting between 30 days before and 30 days after cohort entry start date
  - numeric value between 30 and 60.

with continuous observation of at least 180 days prior and 0 days after event index date, and limit initial events to the **earliest event per person.**

The cohort end date will be offset from the index event's start date plus 365 days.

**3. Patients with chronic kidney disease stage 4**

People who have the following:

- Condition occurrence of 'chronic kidney disease' who are ≥ 20 years old.
  - For the first time in the person's history.
- having at least one measurement of 'MDRD GFR'
  - starting between 30 days before and 30 days after cohort entry start date
  - numeric value between 15 and 30.

with continuous observation of at least 180 days prior and 0 days after event index date, and limit initial events to the **earliest event per person.**

The cohort end date will be offset from the index event's start date plus 365 days.

**4. Patients with chronic kidney disease at stage 5**

People who have the following:

- Condition occurrence of 'chronic kidney disease' who are ≥ 20 years old.
  - For the first time in the person's history.
- having at least one measurement of 'MDRD GFR'
  - starting between 30 days before and 30 days after cohort entry start date
  - numeric value less than 15.

with continuous observation of at least 180 days prior and 0 days after event index date, and limit initial events to the **earliest event per person.**

The cohort end date will be offset from the index event's start date plus 365 days.

**5. Patients with end-stage renal disease and dialysis**

People who have the following:

- Condition occurrence of 'chronic kidney disease' who are ≥ 20 years old.
  - For the first time in the person's history.
- having at least one measurement of 'MDRD GFR'
  - starting between 30 days before and 30 days after cohort entry start date
  - numeric value less than 15.

with continuous observation of at least 180 days prior and 0 days after event index date, and limit initial events to the **earliest event per person.**

The cohort end date will be offset from the index event's start date plus 365 days.

Inclusion Criteria

1. with dialysis

Entry events having at least one procedure occurrence of 'dialysis', starting between 0 day after and all days after cohort entry start date.

**Supplementary Material Appendix 2: Concept set definitions**

**1. Chronic kidney diseases**

| Concept Id | Concept Name | Domain | Vocabulary | Excluded | Descendants | Mapped |
| --- | --- | --- | --- | --- | --- | --- |
| 192359 | Renal failure syndrome | Condition | SNOMED | NO | NO | NO |
| 193782 | End-stage renal disease | Condition | SNOMED | NO | NO | NO |
| 443611 | Chronic kidney disease stage 5 | Condition | SNOMED | NO | NO | NO |
| 443612 | Chronic kidney disease stage 4 | Condition | SNOMED | NO | NO | NO |
| 443597 | Chronic kidney disease stage 3 | Condition | SNOMED | NO | NO | NO |
| 443601 | Chronic kidney disease stage 2 | Condition | SNOMED | NO | NO | NO |
| 443614 | Chronic kidney disease stage 1 | Condition | SNOMED | NO | NO | NO |
| 4019967 | Dependence on renal dialysis | Observation | SNOMED | NO | NO | NO |
| 4301680 | Dialysis care | Observation | SNOMED | NO | NO | NO |

**2. MDRD GFR**

| Concept Id | Concept Name | Domain | Vocabulary | Excluded | Descendants | Mapped |
| --- | --- | --- | --- | --- | --- | --- |
| 46236952 | Glomerular filtration rate/1.73 sq M. predicted [Volume Rate/Area] in Serum, Plasma or Blood by Creatinine-based formula (MDRD) | Measurement | LOINC | NO | YES | NO |

**3. Dialysis**

| Concept Id | Concept Name | Domain | Vocabulary | Excluded | Descendants | Mapped |
| --- | --- | --- | --- | --- | --- | --- |
| 4324124 | Peritoneal dialysis | Procedure | SNOMED | NO | YES | NO |
| 46271816 | Management of peritoneal dialysis | Procedure | SNOMED | NO | YES | NO |
| 4156957 | Chronic ambulatory peritoneal dialysis catheter procedure | Procedure | SNOMED | NO | YES | NO |
| 4120120 | Hemodialysis | Procedure | SNOMED | NO | YES | NO |

**4. CKD-MBD medication**

| Concept Id | Concept Name | Domain | Vocabulary | Excluded | Descendants | Mapped |
| --- | --- | --- | --- | --- | --- | --- |
| 19035704 | Calcium carbonate | Drug | RxNorm | NO | YES | NO |
| 951469 | Calcium acetate | Drug | RxNorm | NO | YES | NO |
| 952004 | Sevelamer | Drug | RxNorm | NO | YES | NO |
| 42899476 | Lanthanum | Drug | RxNorm | NO | YES | NO |
| 985247 | Aluminum hydroxide | Drug | RxNorm | NO | YES | NO |
| 19014202 | Alfacalcidol | Drug | RxNorm | NO | YES | NO |
| 19035569 | Calcifediol | Drug | RxNorm | NO | YES | NO |
| 19035631 | Calcitriol | Drug | RxNorm | NO | YES | NO |
| 19095164 | Cholecalciferol | Drug | RxNorm | NO | YES | NO |
| 1512446 | Doxercalciferol | Drug | RxNorm | NO | YES | NO |
| 19045045 | Ergocalciferol | Drug | RxNorm | NO | YES | NO |
| 35198167 | Maxacalcitol | Drug | RxNorm Extension | NO | YES | NO |
| 1517740 | Paricalcitol | Drug | RxNorm | NO | YES | NO |
| 1548111 | Cinacalcet | Drug | RxNorm | NO | YES | NO |

**Supplemental Figure S1: Yearly CKD-MBD medication prescription pattern and treatment pathway for ESKD patients.**

**
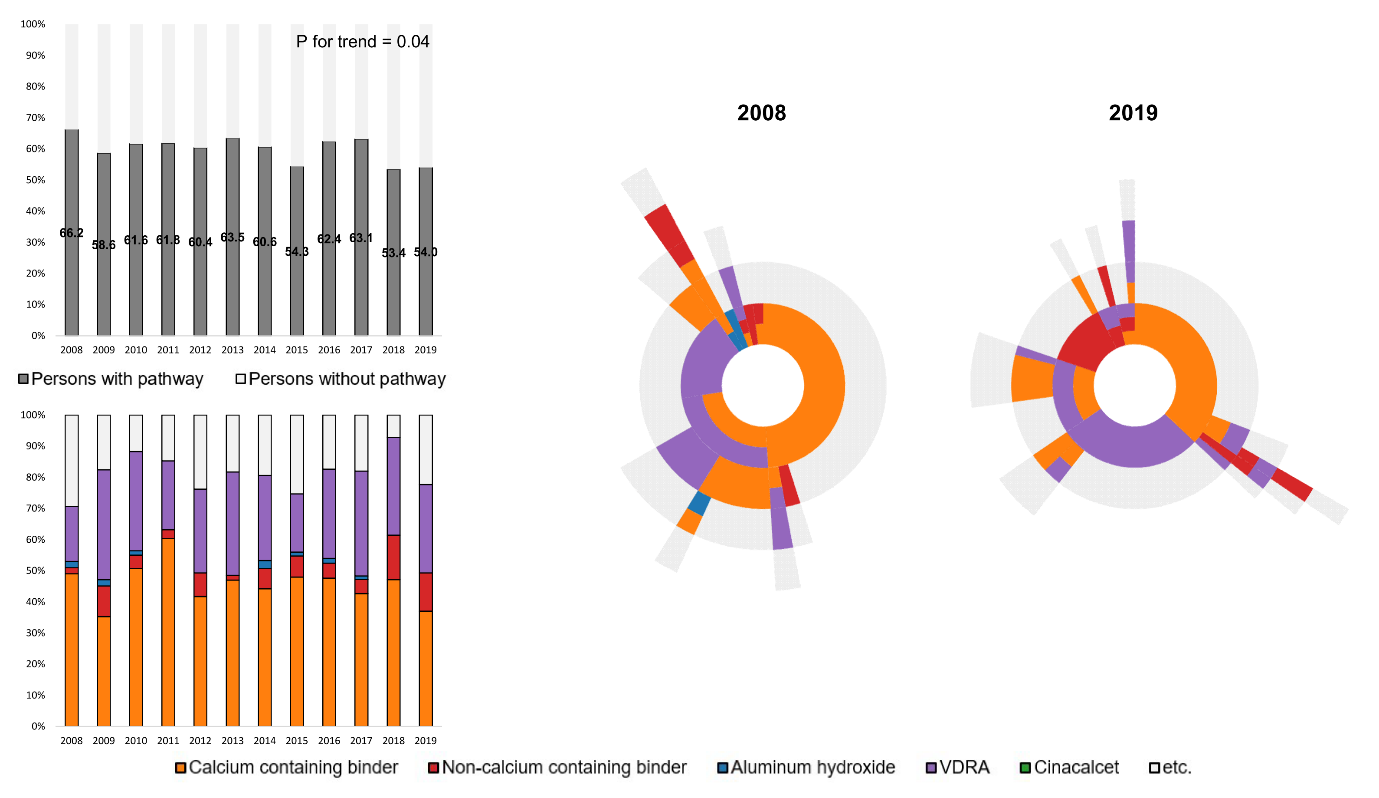
**
